# Androgen Receptor in Laryngeal Carcinoma: Could There Be an Androgen-Refractory Tumor?

**DOI:** 10.5402/2011/180518

**Published:** 2011-11-29

**Authors:** Anastasios K. Goulioumis, John Varakis, Panos Goumas, Helen Papadaki

**Affiliations:** ^1^Department of Anatomy, School of Medicine, University of Patras, Terpsitheas 61, 26442 Patras, Greece; ^2^Otorhinolaryngology-Head and Neck Surgery Department, School of Medicine, University of Patras, Terpsitheas 61, 26442 Patras, Greece

## Abstract

Androgen receptors (ARs) which are implicated in the pathogenesis of several malignancies can also be a possible downstream effector in laryngeal cancer. In the present study, 97 invasive squamous laryngeal carcinomas were studied by immunohistochemistry for protein expression of AR. Androgen receptors were expressed in 52.6% of tumor specimens, suggesting their implication in the pathogenesis of this tumor. Our study's aim was to investigate the hypothetical scenario of an androgen refractory laryngeal carcinoma where androgen receptors can be activated by nodal molecules in the course of an Epithelial-to-mesenchymal transition (EMT) phenomenon. In line with this we correlated AR expression with the expression of ILK, p-Akt, E-cadherin, *β*-catenin in our sample as well as with tumor grade and TNM stage. A reverse correlation between nuclear AR and cytoplasmic ILK expression was evidenced, indicating an interaction of those molecules in laryngeal carcinoma. Finally in our material, in those carcinomas that were expressing ARs, stronger nuclear expression of the receptor was characterized by poorer cell differentiation and correlated with the acquisition of EMT features like E-cadherin loss and *β*-catenin translocation raising a question whether activated ARs can drive an EMT procedure.

## 1. Introduction

Larynx constitutes a hormone-target organ. It is during puberty when under the influence of androgens the larynx of males undergoes anatomical modifications leading to the deepening of voice [1]. While androgens exert this already known developmental function on larynx, it is highly possible that hormones still possess a role in the pathogenesis of carcinomas deriving from this organ, similarly to what happens in other malignancies like prostate cancer [2].

Androgens are steroids functioning through nuclear receptors, which can act as ligand-dependent transcription factors. Prior to ligand binding, the androgen receptors (ARs) are held inactive in the cytoplasm through association with heat shock proteins and are precluded from DNA binding. Ligand binding releases the inhibitory heat shock proteins, and the receptor rapidly translocates to the nucleus, where it binds DNA as a homodimer on androgen responsive elements within the regulatory regions of target genes which are implicated in the cell cycle and apoptosis [3].

Previous studies have documented the expression of ARs in laryngeal carcinoma and investigated their role in laryngeal carcinoma's pathogenesis [4–6]. However, controversial results about the expression of the receptor [7, 8] and failure of antiandrogen therapies designed to eliminate the hormonal level of patients [9] leaded to the early abandonment of efforts for such therapeutical approaches. Besides, epidemiological data concerning laryngeal carcinoma shows a pick incidence of that tumor in an age when a normal reduction of androgen levels in males has already occurred [10, 11]. Thus, androgens do not seem to have a clear participation in laryngeal cancer pathogenesis.

In the case of prostate cancer where androgens are essential for the progression of malignant cells, androgen deprivation is a crucial therapeutic approach [12]. However in the course of prostate cancer development, there is a phase when castrated levels of testosterone fail to inhibit the growth of malignant cells [13], even if they still require androgen receptor activity for their growth.

The molecular basis of the formation of an androgen-refractory cancer implicates mainly AR mutations, AR gene amplification, and expression of coactivators that enhances the AR action [13–15]. It is also possible that ARs can be activated by molecules of signaling transduction pathways that are being activated in malignant cells; for example, it is reported in prostate carcinoma that the molecular pathway activated by Human Epidermal growth factor Receptor-2 (HER2) triggers Src association with ARs [13–15]. In addition, IL-6 phosphorylates factors like STAT-3, MAPK, PI3K/Akt, and Oncostatin M that are found to induce AR activity [13–15]. We hypothesize that a similar condition could take place in the case of laryngeal carcinoma.

Recently we found that integrin-linked kinase (ILK) and p-Akt are overexpressed in laryngeal carcinoma [16]. Both molecules have already been documented to be involved in several processes thought to be critical in carcinogenesis, including cell adhesion, aberrant cell proliferation, evasion from apoptosis, promotion of angiogenesis, and tumor cell invasiveness [17, 18]. The nodal role of ILK and p-Akt in the crosstalk of several molecular pathways, their capability to activate proteins via phosphorylation, and the common molecular characteristics that depend both on these molecules and on androgen receptors prompted us to evaluate their correlation with the expression of ARs in laryngeal cancer.

Currently there is a vast investigation on the way malignant cells acquire a metastatic potential via an epithelial to mesenchymal transition (EMT) [19]. Downregulation of E-cadherin and activation of *β*-catenin in a Wnt pathway manner, that occur in the course of EMT, represents key molecular events in the development and progression of several human malignancies [20], including laryngeal cancer [21, 22]. It is already suggested in the case of prostate cancer that ARs are involved in EMT process [23]. Additionally, ARs and Wnt pathway are shown to interact at many levels [24–26], but no similar studies concerning laryngeal carcinoma are available.

In a series of 97 invasive human laryngeal squamous cell carcinomas we studied by immunohistochemistry the expression of androgen receptors and we correlated them with the expression of ILK, p-Akt (Ser 473), E-cadherin, and *β*-catenin. We additionally correlated our results with clinicopathological parameters such as tumor grade and stage.

## 2. Materials and Methods

### 2.1. Tissue Specimens

The study was performed in accordance with the institutional ethical guidelines and has been approved by the Committee on Research and Ethics and the Scientific Committee of the University Hospital of Patras, Greece. Formalin-fixed, paraffin-embedded tissue samples from 97 primary human invasive squamous laryngeal carcinomas were obtained from the Department of Pathology, Agios Andreas General Hospital, Patras, Greece. Adjacent nonneoplastic laryngeal tissue was used as control. Our material was randomly selected and was consisted of 2 women and 95 men. Ages ranged from 40 to 86 years, with an average age of 60 years. The WHO classification [27] of tumours was used to determine the histological grade: 25/97 tumours (25.8%) were classified as Grade I, 40/97 (41.2%) as Grade II, and 32/97 (33%) as Grade III. Thirty two out of 97 (33%) tumours were stage I, 26/97 (26.8%) stage II, 17/97 (17.5%) stage III, and 22/97 (22.7%) stage IV A according to TNM staging.

### 2.2. Immunohistochemistry

Representative 4 *μ*m tissue sections were dewaxed in xylene and rehydrated in graded ethanols. Antigen retrieval was enhanced by microwaving the slides in 0.01 M citrate buffer (pH = 6). Endogenous peroxidase activity was quenched by treatment with 1% hydrogen peroxide for 25 min, followed by incubation with protein blocking solution. Sections were subsequently incubated with anti-AR (DAKO, Hamburg, Germany, dilution 1 : 20), for one hour in room temperature and primary rabbit anti-ILK (Santa Cruz Biotechnology, CA, USA, dilution 1 : 500), rabbit anti-p-Akt (Ser473) (Cell Signaling, Beverly, MA, dilution 1 : 40), mouse anti-*β*-catenin (BD Biosciences,CA, USA, dilution 1: 2000), and mouse anti-E-cadherin (BD Biosciences, CA, USA, dilution 1 : 2000) overnight at 4°C. Bound primary antibody was detected using the biotin-streptavidin-peroxidase method (Envision Detection Kit, DAKO, Hamburg, Germany) and visualized using diaminobenzidine as the chromogen. Slides were counterstained with haematoxylin, dehydrated and mounted. For negative controls, blocking solution was added instead of the primary antibody.

### 2.3. Immunohistochemical Evaluation

All slides were assessed by one pathologist (H.P.) and one investigator (A.G.) independently and blinded to the case. Both intensity of staining and percentage of positive cells were taken into account and the following scoring system was used: 0: no staining or weak staining in less than 10% of tumor cells, 1: weak staining in 10–40% of tumor cells or moderate staining in <40% of tumor cells, 2: weak staining in >70% of cells, moderate staining in 40–70% of tumor cells or strong staining in 10–40% of tumor cells, and 3: moderate staining in >70% or strong staining in more than 40% of tumor cells. Cases with score 0 were considered negative and cases with scores 1, 2, or 3 were considered positive.

### 2.4. Statistical Analysis

Statistical analysis was performed with the SPSS for Windows, release 15.0 (SPSS Inc., Chicago, IL, USA). Correlations of protein expression levels with clinicopathological parameters were analyzed with the nonparametric Kruskal-Wallis or Mann-Whitney tests for ordinal data and chi-square test for nominal data. Correlations between expression of proteins (immunohistochemical scores) were evaluated by the Spearman rank-order correlation coefficient. Parametric tests were performed with correction for ties. The significance level was defined as *P* < 0.05.

## 3. Results

### 3.1. Androgen Receptors Are Overexpressed in Laryngeal Carcinomas

**Figure 1:**
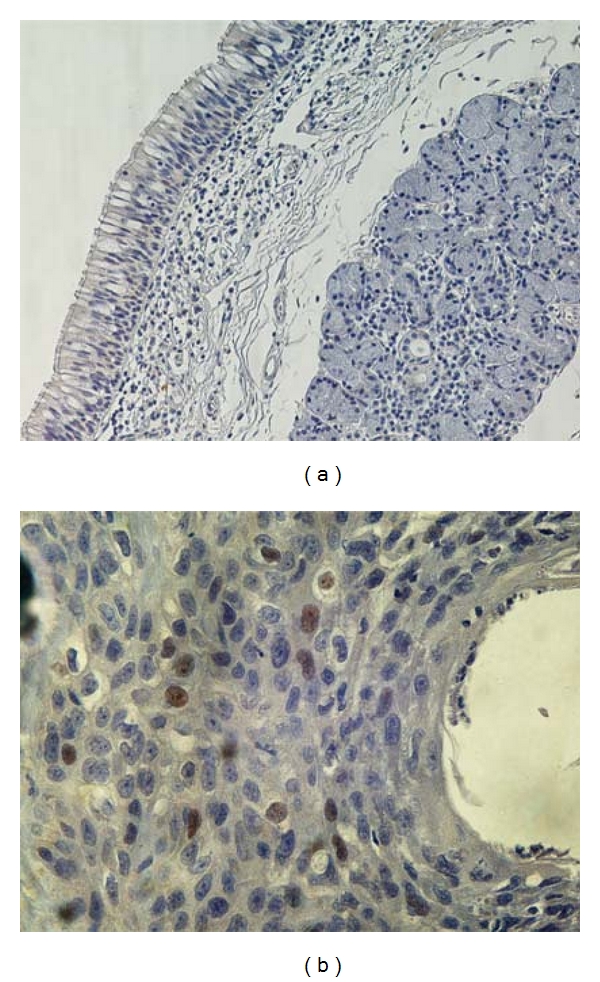
(a) Non-neoplastic laryngeal tissue without AR expression (×200). (b) Nuclear expression of AR in laryngeal carcinoma. (×400).

Immunohistochemical staining for ARs was performed in 97 tumors and adjacent nonneoplastic laryngeal tissue. Immunoreactivity for ARs in adjacent nonneoplastic laryngeal tissue was absent. In contrast 51/97 (52.6%) of laryngeal tumors were positive for ARs (Figure 1). AR expression was confined to the nucleus of cancer cells. In 43.3% of carcinomas nuclear expression of AR was weak, while moderate to strong AR immunostaining was observed in only 9.3% of cases.

We documented a reverse correlation of the expression of ARs in the nucleus with the expression of ILK in the cytoplasm (*r* = −0.260, *P* = 0.010). There was no statistically significant correlation of AR expression with p-Akt, *β*-catenin, E-cadherin (Table 1), or tumour grade and TNM stage (Table 2).

## 4. Discussion

Androgen receptors have a known developmental role in several human malignancies functioning as transcription factors [3]. In the present study we confirmed the expression of ARs in laryngeal carcinoma, as it was already shown by previous studies [4–6].

The existence of an androgen-refractory laryngeal carcinoma could be an interesting hypothesis. According to it ARs may retain their transcription factor activity without the hormone been adapted on them but via activation by molecular pathways that are functioning during EMT. Most of the molecular pathways which are activated during the course of EMT phenomenon intersect to nodal factors like ILK and p-Akt [16] and, among others, conclude to abolishment of E-cadherin from the cell membrane and translocation of *β*-catenin from the membrane to the nucleus [28].

In the current study we did not find any correlation between androgen receptors and p-Akt although it is known in case of prostate cancer that androgen receptors can be activated by the signaling transduction molecular pathway of p-Akt [29, 30], revealing involvement of different molecular pathways in other malignancies.

Additionally, in the current study we show a correlation of low expression of AR in tumors with ILK cytoplasmic overexpression (*r* = −0.260, *P* = 0.01) which possibly suggests an interaction of those molecules in laryngeal cancer, yet not in line with the hypothesis of ARs being activated by ILK. It seems that ILK could oppose the activation of ARs in laryngeal carcinoma and thus their tumor proliferative function. Nevertheless, a tumor suppressive role of ILK has been described in several tumors [17, 31]. We hypothesize that ILK could have a direct association with ARs in the cytoplasm precluding them from activation by other molecular pathways. Such an interaction between ILK and AR has never been described in the literature; however a similar hypothesis has been suggested for another nuclear receptor ER*α* [31].

In our material, androgen receptor expression did not correlate with the expression of *β*-catenin. This finding was rather unexpected since there are studies that have documented multilevel interactions of androgen receptors with Wnt pathway components in other types of carcinomas. Firstly AR can act as cofactors of the *β*-catenin/LEF transcription factor [24, 25]. Additionally, androgen receptors are transcription targets of *β*-catenin/LEF transcription factor [24]. Finally, ARs can potentially be activated either by *β*-catenin being adapted on them [21] or by GSK-3b phosphorylation [24]. Nevertheless, it is commendable that when we excluded the cases that had negative AR expression we documented a statistically significant correlation between the intensity of AR expression, the abolishment of *β*-catenin from the membrane (*r* = −0.762, *P* = 0.017), and its translocation to the nucleus (*r* = 0.791, *P* = 0.011). In those selected cases we also show a correlation between intense nuclear expression of AR and abolishment of E-cadherin from the membrane (*r* = −0.671, *P* = 0.048).

In our total sample we failed to correlate AR expression with clinicopathological parameters; yet, in the selected cases characterized by any detection of AR nuclear expression, we documented a correlation of intense AR expression with high Grade tumors (*r* = 0.707, *P* = 0.033).

In conclusion, taking into account the limitations of our study, such as small sample size, descriptive character and lack of genomic, and transcriptional and posttranscriptional analysis, we evidenced androgen receptor expression in laryngeal cancer suggesting a possible role in the pathogenesis of that malignancy. We investigated the hypothetical scenario of an androgen refractory laryngeal carcinoma where androgen receptors can be activated by nodal molecules in the course of EMT. We suggest that ILK among others could possibly exert a role in such a molecular crosstalk; however further research is needed to support that consumption. Finally in our material interestingly in those carcinomas that were expressing ARs, stronger nuclear expression of the receptor was characterized by poorer cell differentiation and correlated with the acquisition of EMT features like E-cadherin loss and *β*-catenin translocation raising a question whether activated ARs can contribute together with other molecular pathways to the development of an Epithelial-Mesenchymal Transition in the course of laryngeal carcinoma metastasis. Further investigation is needed to evaluate AR and the molecular pathways of cancer that can activate it, as potential molecular targets for laryngeal carcinoma therapy.

## Figures and Tables

**Table 1:** Correlation of Androgen receptors expression with E-cadherin, *β*-catenin, p-Akt, and ILK expression in laryngeal carcinoma by the Spearman rank-order correlation coefficient.

	Membranous E-cadherin	Membranous *β*-catenin	Cytoplasmic *β*-catenin	Nucleus *β*-catenin	Cytoplasmic p-Akt	Nucleus p-Akt	Cytoplasmic ILK
Nuclear	*r* = −.067	*r* = .065	*r* = .000	*r* = .113	*r* = −.099	*r* = .100	*r* = −.260
AR	*P* = 0.515	*P* = 0.527	*P* = 0.999	*P* = 0.270	*P* = 0.332	*P* = 0.332	*P*^a = 0.010

^a^ *P* < 0.05 was considered statistically significant.

**Table 2:** Androgen receptors expression in laryngeal carcinoma. There is correlation with clinicopathological parameters.

Nuclear Androgen Receptor^a^						
	N	0 *n* (%)	1 *n* (%)	2 *n* (%)	3 *n* (%)	*P* value^b^

Carcinomas						

total	97	46 (47.4)	42 (43.3)	6 (6.2)	3 (3.1)	

Grade						0.852

I	25	13 (52)	10 (40)	2 (8)	0 (0)	

II	40	19 (47.5)	16 (40)	4 (10)	1 (2.5)	

III	32	14 (43.8)	16 (50)	0 (0)	2 (6.2)	

TNM Stage						0.890

I	32	13 (40.6)	17 (53.2)	1 (3.1)	1 (3.1)	

II	26	14 (53.8)	8 (30.8)	2 (7.7)	2 (7.7)	

III	17	9 (53)	7 (41.1)	1 (5.9)	0 (0)	

IV	22	10 (45.5)	10 (45.5)	2 (9)	0 (0)	

^a^AR expression was scored as described in methods. ^b^Kruskal-Wallis or Mann-Whitney test; *P* < 0.05 was considered statistically significant.
